# Healthcare worker infection with SARS-CoV-2 and test-based return to work

**DOI:** 10.1017/ice.2020.438

**Published:** 2020-08-26

**Authors:** Erica S. Shenoy, Lauren R. West, David C. Hooper, Rosemary R. Sheehan, Dean Hashimoto, Ellyn R. Boukus, Marisa N. Aurora, Dustin S. McEvoy, Michael Klompas

**Affiliations:** 1 Division of Infectious Diseases, Massachusetts General Hospital, Boston, Massachusetts; 2 Infection Control Unit, Massachusetts General Hospital, Boston, Massachusetts; 3 Department of Medicine, Harvard Medical School, Boston, Massachusetts; 4 Human Resources, Mass General Brigham, Boston, Massachusetts; 5 Occupational Health Services, Mass General Brigham, Boston, Massachusetts; 6 Data and Analytics Organization, Mass General Brigham, Boston, Massachusetts; 7 Clinical Informatics, Mass General Brigham, Boston, Massachusetts; 8 Department of Population Medicine, Harvard Medical School and Harvard Pilgrim Health Care Institute, Boston, Massachusetts; 9 Department of Medicine, Brigham and Women’s Hospital, Boston, Massachusetts

Infection of healthcare workers (HCWs) with SARS-CoV-2 can result from either community or workplace exposure. Determination of when the HCW can return to work (RTW) has important implications for patient and workforce safety as well as workforce preservation. On April 13, 2020, the Centers for Disease Control and Prevention (CDC) modified its guidance to indicate a preference for the use of a test-based strategy to determine when HCWs may return to work in healthcare settings over a symptom-based strategy. Subsequent iterations have indicated that either time plus symptom–based or test-based approaches are acceptable.^1^ At Massachusetts General Brigham (MGB), test-based RTW criteria was established at the start of the coronavirus disease 2019 (COVID-19) pandemic. We report average intervals until test-based clearance and the number of excess lost work days using test-based clearance.

## Methods

The MGB system is a not-for-profit healthcare system with 78,000 employeees, 2 academic health centers, 6 community hospitals, 2 speciality hospitals, a rehabilitation network, as well as urgent care centers, community health centers, and home-based care programs. Employees with symptoms compatible with COVID-19 were referred to MGB Occupational Health Services for evaluation and were referred for nasopharyngeal (NP) sampling. Various viral RNA nucleic acid amplification methods were used (Supplementary Material online). Initially, testing was contingent upon symptom onset with respect to clinical duties. As testing capacity expanded, all employees with any symptoms consistent with COVID-19 were referred for testing.

The following RTW criteria were implemented: resolution of fever without fever-reducing medications, improvement in respiratory symptoms, and at least 2 consecutive negative NP swabs collected ≥24 hours apart. A minimum interval of time from resolution of symptoms to first test of clearance was not specified.

Outcomes included number of days to first and second sequential negative NPs summarized using mean, median, standard deviation, Kaplan Meier estimator, and confidence intervals. Lost work days were calculated comparing a time plus symptom–based clearance to the test-based protocol. For the former, we assumed that the day the employee was tested under test-based clearance indicated the resolution of symptoms. All analyses were completed in R version 4.0.0 statistical software (R Foundation for Statistical Computing). The activities conducted here were considered routine infection control and occupational health procedures and not human subjects research by the institutional review board.

## Results

Between March 7, 2020, and April 22, 2020, 8,930 employees were tested and 1,049 (11.7%) were positive for SARS-CoV-2. Of those, 37 (3.5%) were hospitalized at an MGB institution within 7 days of their positive test.

**Fig. 1.**
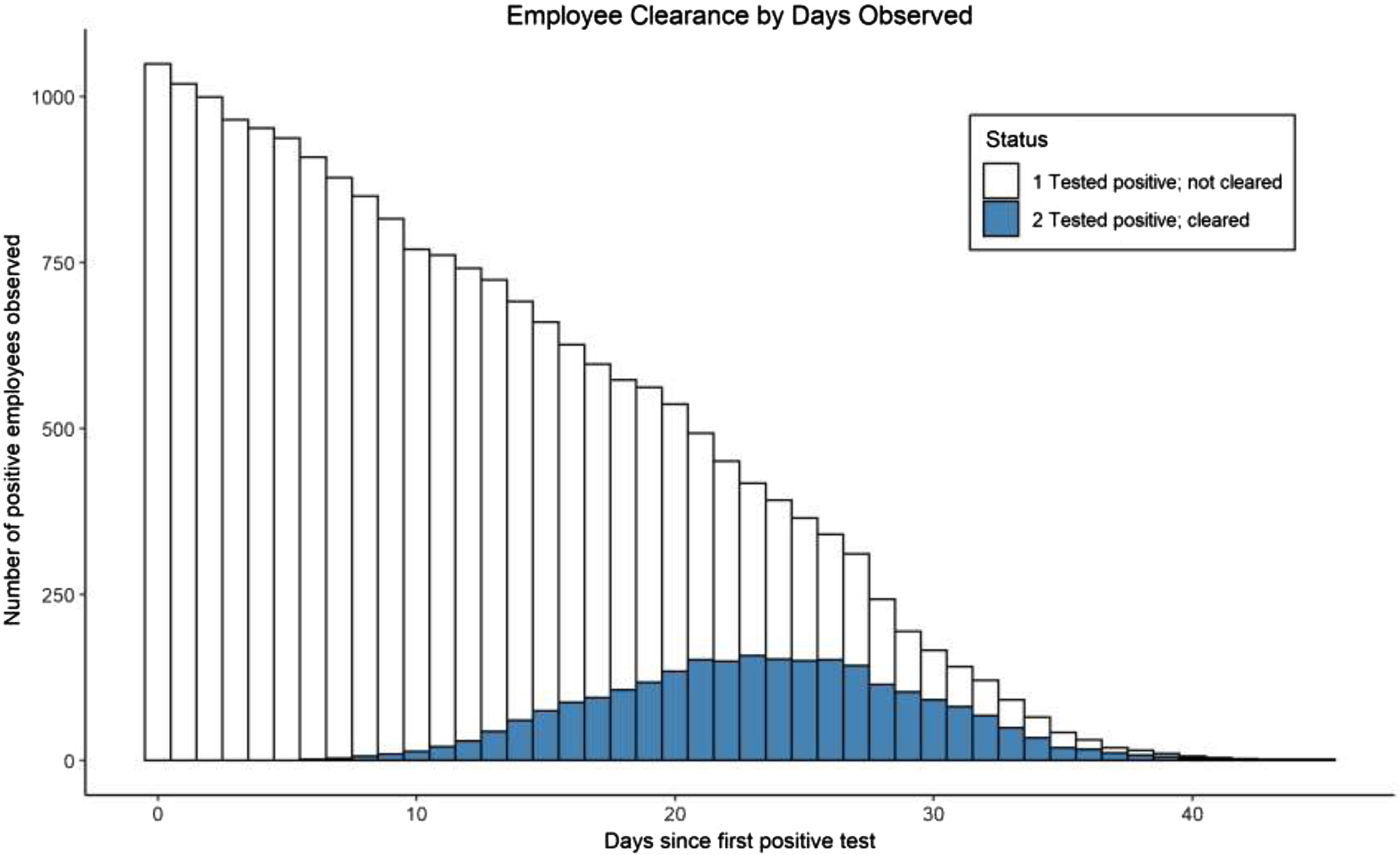
Employee return to work (RTW) by days observed. Employees in whom 2 sequential negative nasopharyngeal swabs were obtained at least 24 hours apart are shown in blue. Those without repeat testing or with a single negative swab are shown in white.

Among 590 HCWs with subsequent testing, 425 (72.0%) had at least 1 negative NP swab (Supplementary Fig. S1 online). The mean and median number of days from first positive to first negative were 17.1 (SD, 6.7) and 17 (IQR, 9), with a minimum of 2 days and a maximum observed of 38 days. Of the 425 HCWs with positive SARS-CoV-2 test results, 263 (61.9%) had a sequential second negative NP. The mean and median number of days from first positive to second negative were 19.5 (SD, 6.1) and 19 (IQR, 8), with a minimum observed of 6 days, 25th percentile at 15 days, and a maximum observed at 37 days (Fig. 1). The Kaplan-Meier estimate of median time to clearance was 29 days (95% CI, 28–31) (Supplementary Fig. S2 online). We estimated that test-based clearance accounted for an additional 4,097 days of cumulative lost work time, corresponding to a mean of 7.2 additional days of work lost per employee than would have been accrued using the time plus symptom-based clearance method.

## Discussion

The HCWs diagnosed and treated for COVID-19 had prolonged recovery of viral RNA; the test-based strategy resulted in a median time to RTW of 19 days. The long duration of PCR positivity is consistent with prior studies. The time plus symptom–based criteria would have resulted in 4,097 fewer lost work days, or an average of 7.2 fewer days of work lost per employee. The additional psychological toll of prolonged positivity on HCW well-being was not assessed; some HCWs reported stress and anxiety from isolating within their households and extended delays in returning to work.

This research had several limitations. A subset of employees were still in process for RTW considerations at the end of the study period. Some employees lost to follow-up include those who elected not to be retested despite meeting criteria, including those who were working remotely during the study period.

Also during the study period, additional evidence emerged regarding lack of transmission after recovery from symptoms,^2–6^ which has informed a shift away from a test-based strategy in favor of a time plus symptom–based strategy for ending isolation and permitting RTW in healthcare settings. Viral load has been shown to be highest at the time of symptom onset and then to decline within a week thereafter.^4^ Transmission is rare among close contacts of COVID-19 cases when that contact occurred after day 6 of the source individual’s infection,^3^ and transmission has not been reported from close contacts of patients who have tested positive after recovery from their illness.^5^ These observations were noted by the CDC in their May 3, 2020, decision memo supporting a move away from test-based strategies for discontinuation of isolation.^7^ MGB accordingly switched to time plus symptom–based RTW criteria on May 22, 2020.

In summary, persistently positive RNA PCRs are common in healthcare workers and present a formidable challenge to healthcare institutions.^8^ If test-based criteria are used for RTW, we recommend establishing a minimum duration of days prior to test of clearance. Switching to time plus symptom–based clearance criteria will allow an ealier RTW for most workers and can aid in workforce preservation.

## Supplementary material

For supplementary material accompanying this paper visit http://dx.doi.org/10.1017/ice.2020.438. click here to view supplementary material
