# Supplementary material for: Healthcare worker infection with SARS-CoV-2 and test-based return to work
Source: Infect Control Hosp Epidemiol. 2020 Aug 26:1–3. doi: 10.1017/ice.2020.438 (PMC7484303; doi:10.1017/ice.2020.438)

Healthcare Worker Infection with SARS-CoV-2 and Test-Based Return to Work

Erica S. Shenoy, MD, PhD^1, 2, 3 †^

Lauren R. West, MPH^2^

David C. Hooper, MD^1, 2, 3^

Rosemary R. Sheehan^4^

Dean Hashimoto, MD^5^

Ellyn R. Boukus, MA^6^

Marisa M. Aurora, MPH^6^

Dustin S. McEvoy^7^

Michael Klompas, MD, MPH^8,9^

^1^ Division of Infectious Diseases, Massachusetts General Hospital, Boston, MA

^2^ Infection Control Unit, Massachusetts General Hospital, Boston, MA

^3^ Department of Medicine, Harvard Medical School, Boston, MA

^4^ Human Resources, Mass General Brigham, Boston, MA

^5^ Occupational Health Services, Mass General Brigham, Boston, MA

^6^Data and Analytics Organization, Mass General Brigham, Boston, MA

^7^Clinical Informatics, Mass General Brigham, Boston, MA

^8^ Department of Population Medicine, Harvard Medical School and Harvard Pilgrim Health Care Institute, Boston, MA

^9^ Department of Medicine, Brigham and Women’s Hospital, Boston, MA

**Keywords**: SARS-CoV-2, COVID-19, healthcare worker infection

^†^ Corresponding author: Erica S. Shenoy, MD, PhD

55 Fruit Street, BUL-3-334

Boston, MA 02114

Tel: 617-643-5637

eshenoy@mgh.harvard.edu

**Supplement.**

**SARS-CoV-2 Viral RNA Detection Methods**

During the course of the study, the following assays were in use across MGB.

- TaqPath COVID-19 Combo Kit (ThermoFisher, Waltham, MA)
- Panther Fusion® SARS-CoV-2 Assay (Hologic, Marlborough, MA)
- CRSP SARS-CoV-2 Real-time Reverse Transcriptase (RT)-PCR Diagnostic Assay (Broad Institute, Cambridge, MA)
- cobas® SARS-CoV-2 Test (Roche Diagnostics Corporation, Indianapolis, IN)
- Xpert® Xpress SARS-CoV-2 (Cepheid, Sunnyvale, CA)
- Abbott RealTi*m*e SARS-CoV-2 (Abbott Laboratories, Abbott Park, IL)
- Massachusetts Department of Public Health State Lab

**Figure S1 Legend. Employee Testing Outcomes.** Note that detailed outcomes after indeterminate results were obtained are not shown, nor are subsequent testing results in retest groupings shaded in grey. NP: nasopharyngeal swab.


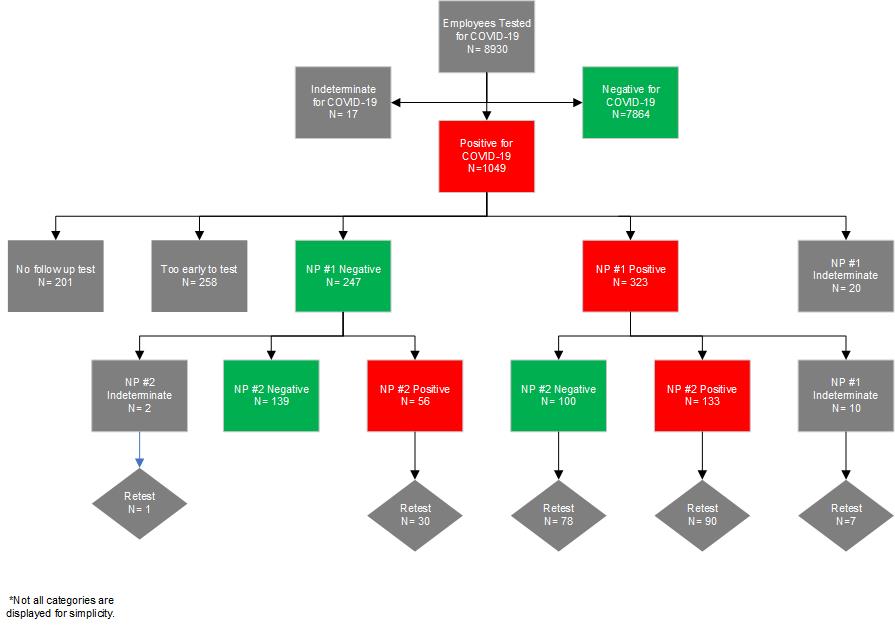


**Figure S2 Legend.** **Kaplan-Meier Estimate of Time to Employee RTW.** Clearance defined as time to two sequential negative nasopharyngeal swabs, obtained at least 24 hours apart.


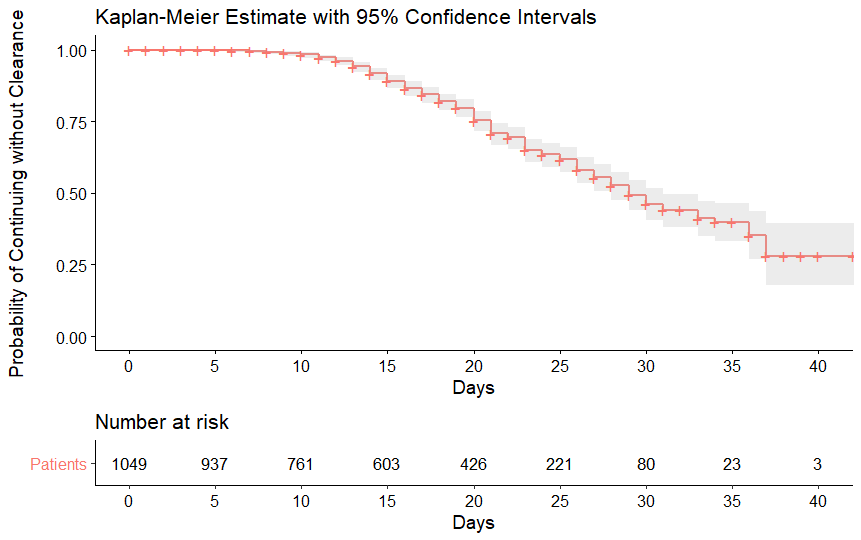

Supplement: Supplementary file 1 [file S0899823X20004389sup001.docx]
